# Comparison of Antipsychotics for the Treatment of Patients With Delirium and QTc Interval Prolongation: A Clinical Decision Analysis

**DOI:** 10.3389/fpsyt.2021.609678

**Published:** 2021-06-25

**Authors:** Ken Kurisu, Kazuhiro Yoshiuchi

**Affiliations:** Department of Stress Sciences and Psychosomatic Medicine, Graduate School of Medicine, The University of Tokyo, Tokyo, Japan

**Keywords:** delirium, QTc prolongation, antipsychotics, clinical decision analysis, quetiapine

## Abstract

**Background:** Antipsychotics are frequently used to treat delirium but often induce corrected QT (QTc) prolongation, which can be lethal by causing torsade de pointes. Nonetheless, the selection of antipsychotics to treat delirium patients with prolonged baseline QTc intervals remains unclear. We aimed to assess the utility of antipsychotics based on their effects on treatment outcomes and QTc intervals.

**Methods:** A clinical decision analysis was conducted using data on the effects of antipsychotics on treatment outcomes and QTc intervals from published network meta-analyses. We quantified the utility of six antipsychotics (amisulpride, haloperidol, olanzapine, quetiapine, risperidone, and ziprasidone) using a decision tree and the obtained effect sizes. Subsequently, we conducted sensitivity analyses using multiple utility settings and another dataset. We also performed a probabilistic sensitivity analysis using Monte Carlo simulation, in which the effects of antipsychotics were randomly sampled given the plausible range.

**Results:** Amisulpride showed the highest utility when the baseline QTc interval was 420 ms. Quetiapine showed the highest utility when the baseline QTc interval was ≥450 ms. The sensitivity analyses also showed the superiority of quetiapine when the baseline QTc intervals were prolonged.

**Conclusions:** Decision analysis suggests that quetiapine is the optimal antipsychotic drug for the treatment of patients with delirium and prolonged baseline QTc intervals.

## Introduction

Delirium is an acute psychiatric disorder common among hospitalized patients, and the short-term use of small doses of antipsychotics is the treatment of choice (1). Corrected QT (QTc) prolongation is a serious adverse effect of antipsychotics (2). QTc prolongation leads to torsade de pointes (TdP), which can cause death (3, 4). Clinicians should prescribe antipsychotics with caution, especially in patients with prolonged baseline QTc intervals. Several types of antipsychotics reportedly cause no significant QTc prolongation in treating delirium (5–8), whereas other reports have revealed that they elevate the risk of QTc prolongation and TdP during delirium treatment (9–12). However, few studies have analyzed the usefulness of antipsychotics using treatment effects and effects on QTc prolongation simultaneously. There is little evidence for selecting antipsychotics to treat delirium patients with prolonged baseline QTc intervals, and research is required to determine the optimal antipsychotic drug for such cases.

Decision analysis, a mathematical method that can compare multiple treatment options using data from published literature, has been widely applied in clinical fields (13–16). Specifically, the method calculates the utility of treatments based on transition probabilities (e.g., response rates of treatment drugs) and utility for disease status (e.g., utility value for improved or non-improved status). The analysis can concurrently use data on adverse events and treatment effects. The usual statistical or machine learning models generally handle only one outcome and cannot perform such simultaneous considerations. We hypothesized that a decision analysis could determine the optimal antipsychotic drug for treating delirium patients with QTc prolongation by considering their effects on delirium treatment and QTc intervals concurrently.

## Materials and Methods

### Model Structure

**Figure 1:**
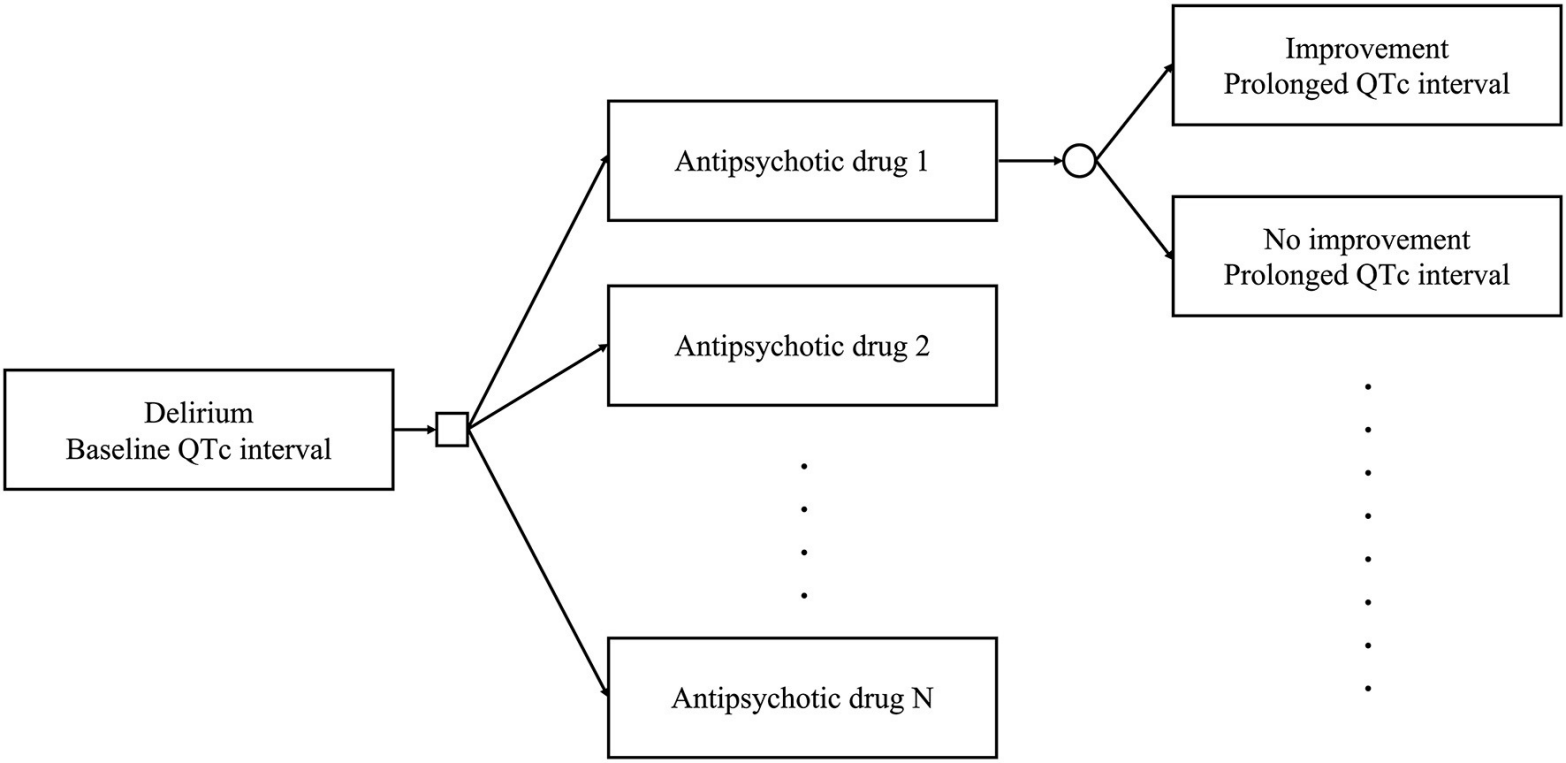
Decision tree used in this study. Clinicians can select an antipsychotic drug among multiple options to treat delirium. The square represents a decision node. The delirium status improves or does not. The QTc interval changes according to the effects of the selected antipsychotic drug. The circle represents a chance node.

We constructed a decision tree to identify the optimal antipsychotic drug for the treatment of patients with delirium and prolonged QTc intervals (Figure 1). The model allows clinicians to select an antipsychotic drug to treat delirium. The square represents a decision node in which clinical decisions are performed. Subsequently, delirium improves or fails to improve, based on the transition probability of the selected antipsychotic drug. The QTc interval also changes based on the effect of the selected antipsychotic drug. The circle represents a chance node, where the status changes according to the decision. Each status has utility based on the delirium status and QTc interval. This model allows us to calculate the utility of antipsychotics. The following analyses were conducted using R (version 4.0.4) (R Foundation for Statistical Computing, Vienna, Austria, 2021).

### Data Sources

We searched for studies on network meta-analysis that can integrate effect sizes from multiple studies (17). We considered network meta-analysis to be suitable as a data source because various types of antipsychotics are used clinically. The PubMed database was explored, and literature published before December 10, 2020, was included in the study.

To gather the transition probabilities of delirium status, we used the following search formula: “(delirium [Title]) AND (‘network meta-analysis’ OR ‘multiple treatment meta-analysis’).” The search yielded three published network meta-analyses that synthesized the therapeutic effects of antipsychotics for delirium using multiple randomized clinical trials (RCTs) (18–20). Wu et al. synthesized the effect sizes of seven types of single-use antipsychotics independently (18). Burry et al. classified antipsychotics into two types (typical or atypical) and compiled their effect sizes (19); however, this classification was unsuited to our aim of comparing antipsychotics independently. Kim et al. performed a stratified analysis for each clinical setting (intensive care unit [ICU], palliative care unit [PCU], and general ward/medical inpatient) (20), which was preferred for sensitivity analysis. Therefore, our main analysis used the data published by Wu et al.: a network meta-analysis of 20 RCTs that included 1,435 participants (18). In the sensitivity analysis, we used the data published by Kim et al.: a network meta-analysis of 24 RCTs that included 1,846 participants (20).

Because these studies integrated odds ratios (ORs) as a therapeutic effect (18, 20), we translated the ORs into transition probabilities. We defined p as the transition probability of the selected drug and p_0_ as that of the placebo. The ORs were translated into transition probabilities according to the following equation: According to a placebo-controlled randomized control study (21), we set p_0_ at 0.5 on day 3 of treatment.

$$\begin{array}{l} \text{OR}=\;\frac{\text{p}/\left(1-\text{p}\right)}{{\text{p}}_{0}/\left(1-{\text{p}}_{0}\right)}\;\Leftrightarrow \text{ p}=\;\frac{\text{A}}{1+\text{A}}\;\left(\text{A}=\text{OR }\cdot \;\frac{{\text{p}}_{0}}{1-{\text{p}}_{0}}\right) \end{array}$$

Subsequently, we used the following search formula to obtain the effects of antipsychotics on QTc prolongation: “(delirium [Title]) AND (‘network meta-analysis’ OR ‘multiple treatment meta-analysis’) AND (QT OR QTc).” As this search strategy identified no appropriate study, we used the following search formula instead: “(antipsychotics OR antipsychotic) AND (‘network meta-analysis’ OR ‘multiple treatment meta-analysis’) AND (QT OR QTc OR tolerability OR safety OR ‘side effect’ OR ‘side effects’).” We found a network meta-analysis synthesizing the effect sizes of antipsychotics on QTc prolongation as continuous variables (i.e., values of prolongation). The study included 51 RCTs investigating the acute treatment of 15,467 participants with schizophrenia (22). To the best of our knowledge, there is little evidence for the heterogeneity of the effects of antipsychotics on QTc prolongation between delirium and schizophrenia. Therefore, we used the data from this study. Although several studies found by the search analyzed the incidence of QTc prolongation (i.e., integrating odds ratios or risk ratios), we considered these studies unsuitable for stratified analyses according to baseline QTc intervals.

In the following analysis, we compared the utility of placebo and six single-use antipsychotics (amisulpride, haloperidol, olanzapine, quetiapine, risperidone, and ziprasidone) included in the two selected studies (18, 22). Table 1 shows the transition probabilities and the effects on QTc intervals obtained from these studies. The plausible range was determined according to the 95% confidence intervals (CIs).

**Table 1:** Effect size obtained from the literature (18, 22).

	**Transition probability to improved status (plausible range)**	**Effect size on QTc interval prolongation (plausible range)**
Amisulpride	0.804 (0.153–0.989)	14.10 (7.71–20.45)
Haloperidol	0.703 (0.510–0.844)	1.69 (−0.23–3.64)
Olanzapine	0.711 (0.415–0.896)	4.29 (1.91–6.68)
Quetiapine	0.791 (0.355–0.963)	3.43 (0.94–6.00)
Risperidone	0.611 (0.359–0.814)	4.77 (2.68–6.87)
Ziprasidone	0.743 (0.324–0.945)	9.70 (7.43–12.04)

*The transition probability to improved status was calculated using the odds ratio obtained from the literature. The transition probability of the placebo was fixed at 0.5. The plausible range corresponds to the 95% confidence interval presented in the literature*.

### Utility Settings

We prepared the following utility function considering the risk attitude to calculate the utility according to QTc intervals (23–26). Because a QTc interval exceeding 500 ms increases the risk of TdP (3, 4), we considered that the utility function should be a decreasing function with a second derivative <0 when the QTc interval is <500 ms and a decreasing function with a second derivative >0 when the QTc interval is >500 ms. To achieve this assumption, we used a sigmoid function with three parameters: minimum utility (Min), maximum utility (Max), and slope of the sigmoid function (Slope).

$$\begin{array}{l} \text{Utility}\left(\text{QTc}\right)\;=\text{ Min }+\;\frac{\left(\text{Max }-\text{ Min}\right)}{1+\text{exp}\left(\text{Slope }\cdot \;\left(\text{QTc }-\;500\right)\right)} \end{array}$$

Each parameter was determined in the baseline settings as follows: For the status with delirium improvement, we fixed the maximum utility at 100 and set the minimum utility at 30. For the status without delirium improvement, we set the maximum utility at 30 and fixed the minimum utility at 0. The slope of the sigmoid function was set to 0.05. Table 2 shows the utility used in the baseline setting.

**Table 2:** Default utility values based on a sigmoid function.

**QTc interval**	**With delirium improvement**	**Without delirium improvement**
–∞	100 (fixed)	30
420	99	29
450	95	28
480	81	22
510	56	11
540	38	4
570	32	1
∞	30	0 (fixed)

*We fixed the maximum and minimum values for the status with delirium improvement and without delirium improvement, respectively. Other values were changed in the sensitivity analysis*.

### Utility Calculation

The QTc interval was calculated as the sum of the baseline value and the effect size of the selected antipsychotic drug. Subsequently, the expected utility was calculated using the following equation:

$$\begin{array}{r} \text{Expected utility }={\text{p }\cdot \text{ Utility}}_{\text{improved}}\left(\text{QTc}\right)+\\ \left(1-\text{p}\right)\;\cdot \;{\text{Utility}}_{\text{unimproved}}\left(\text{QTc}\right) \end{array}$$

We then compared the utility and determined the optimal antipsychotic drug according to the baseline QTc interval.

### Sensitivity Analysis for the Utility Settings

We performed a sensitivity analysis that altered utility settings. First, we prepared multiple parameter patterns for the utility function. Further, we used the following variations of utility function considering risk attitude: a linear function with a second derivative of 0, an exponential function with a second derivative >0, and an exponential function with a second derivative <0 (23–26). The details of these parameters and functions are provided in the Supplementary Material.

### Probabilistic Sensitivity Analysis

We performed a probabilistic sensitivity analysis using Monte Carlo simulation. The transition probabilities and the effects on QTc intervals were randomly sampled 10,000 times from the normal distribution that had a 95% CI determined via the plausible range, and then the utility was calculated using these values. We also performed this analysis by using the different types of utility functions.

### Sensitivity Analysis Using Another Dataset

We performed a sensitivity analysis using the data from the literature by Kim et al. (20). Because this study integrated the effect sizes separately for each clinical setting (ICU, PCU, and general ward), we also performed a decision analysis for each clinical setting. The transition probabilities obtained from this study are listed in Supplementary Table 1.

## Results

### Main Analysis

**Figure 2:**
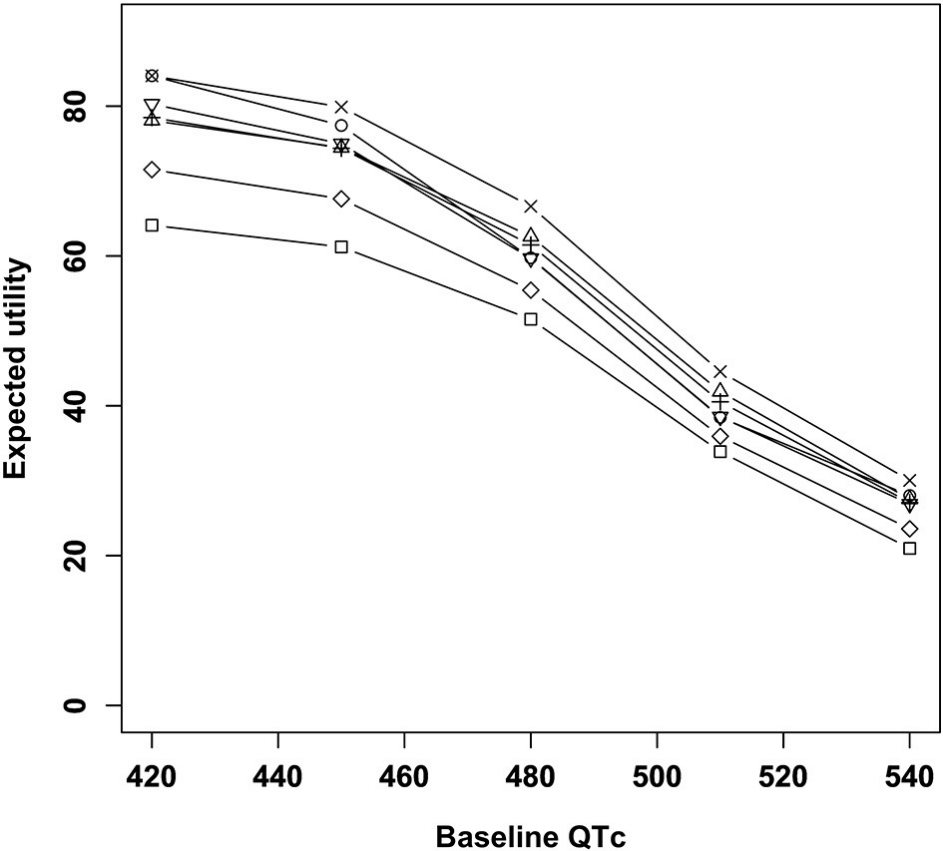
Expected utility of antipsychotics according to baseline QTc intervals. □, Placebo; ○, Amisulpride; Δ, Haloperidol; +, Olanzapine; ×, Quetiapine; ◇, Risperidone; ∇, Ziprasidone.

The results of the main analysis are presented in Figure 2. Amisulpride showed the highest utility when the baseline QTc interval was 420 ms. Quetiapine showed the highest utility when the QTc interval was ≥450 ms. Among the six antipsychotics, risperidone had the lowest utility, and the remaining three drugs had nearly the same utility. The utility of the placebo was lower than that of other antipsychotics.

### Sensitivity Analysis

The ranking of the utility of each antipsychotic drug did not change appreciably despite altering the utility parameters and the utility functions (Supplementary Figures 2–5). In most settings, quetiapine showed the highest utility when the QTc interval was prolonged. In several settings, amisulpride had a higher utility than quetiapine, even when the QTc interval was prolonged.

The results of the probabilistic sensitivity analysis are presented in Table 3. Amisulpride showed the highest utility in more simulations (36%) than the other drugs when the baseline QTc interval was 420 ms. Quetiapine showed the highest utility in more simulations (36–47%) than the other drugs when the QTc interval was ≥450 ms. Other antipsychotics showed the highest utility in a relatively small percentage (at most 18%) of the simulations. Only haloperidol had a higher utility than placebo in over 95% of the simulations. The results did not drastically change when the different utility functions were used (Supplementary Table 2).

**Table 3:** Results of probabilistic sensitivity analysis.

	**Baseline QTc intervals**			
**Antipsychotics**	**420**	**450**	**480**	**510**
**Placebo**				
Utility (fixed)	64.10	61.21	51.55	33.88
Highest utility	0%	0%	0%	0%
**Amisulpride**				
Utility, mean (SD)	78.65 (16.18)	72.32 (15.21)	55.59 (12.70)	35.47 (9.43)
Highest utility	**36%**	28%	13%	10%
Higher than placebo	80%	77%	68%	64%
**Haloperidol**				
Utility, mean (SD)	77.61 (5.99)	73.99 (5.78)	62.18 (5.11)	41.58 (3.90)
Highest utility	5%	7%	14%	18%
Higher than placebo	98%	98%	97%	96%
**Olanzapine**				
Utility, mean (SD)	77.38 (8.70)	73.32 (8.36)	60.55 (7.28)	39.86 (5.50)
Highest utility	9%	11%	14%	15%
Higher than placebo	92%	91%	88%	85%
**Quetiapine**				
Utility, mean (SD)	81.52 (11.26)	77.45 (10.82)	64.48 (9.46)	42.97 (7.15)
Highest utility	31%	**36%**	**46%**	**47%**
Higher than placebo	91%	90%	89%	88%
**Risperidone**				
Utility, mean (SD)	71.06 (8.27)	67.18 (7.93)	55.05 (6.87)	35.65 (5.16)
Highest utility	2%	2%	3%	2%
Higher than placebo	79%	77%	70%	64%
**Ziprasidone**				
Utility, mean (SD)	78.31 (11.36)	73.10 (10.78)	58.07 (9.11)	37.35 (6.77)
Highest utility	18%	16%	11%	8%
Higher than placebo	87%	85%	77%	72%

*The bold text reflects the largest percentage of simulations in which an antipsychotic drug had the highest utility. The table also shows the percentage of simulations in which each drug had a higher utility than the placebo*.

The details of the results of the sensitivity analysis using another dataset are shown in Supplementary Figure 6 and Supplementary Table 3. The utility of quetiapine remained superior to that of haloperidol, ziprasidone, and placebo in ICU settings. In PCU settings, no data on quetiapine were available, and placebo showed a higher utility than haloperidol and risperidone. In general ward settings, haloperidol and quetiapine showed nearly the same utility, which was higher than that of olanzapine, risperidone, and placebo. In the probabilistic sensitivity analysis for general ward settings, haloperidol showed the highest utility in a slightly higher percentage of the simulations than quetiapine.

## Discussion

We performed a clinical decision analysis to assess the optimal antipsychotic drug for treating patients with delirium and QTc prolongation using data from published network meta-analyses. Of the six single-use antipsychotics, amisulpride showed the highest utility when the baseline QTc interval was within the normal range, and quetiapine showed the highest utility when the baseline QTc interval was prolonged. The sensitivity analyses yielded nearly the same results. The use of another dataset showed that quetiapine still had a high utility in ICU and general ward settings, whereas haloperidol showed a slightly higher utility than quetiapine in general ward settings.

Overall, quetiapine showed a high utility among single-use antipsychotics when the baseline QTc interval was prolonged. In the network meta-analyses used in the present study, amisulpride and quetiapine had relatively large ORs for delirium treatment, and the effect of amisulpride on QTc prolongation was larger than that of quetiapine (18, 22). These data may explain the high utility of quetiapine. However, there were no data for quetiapine in PCU settings (20), and the utility of quetiapine was slightly lower than that of haloperidol in general ward settings. A previous review indicated that quetiapine could induce TdP in high-risk patients, such as those with cardiac diseases or electrolyte disturbances (12). Therefore, further investigations, such as analyses stratified according to clinical settings or comorbidities, are required to validate the superiority of quetiapine for delirium patients with prolonged QTc intervals.

Haloperidol had a higher utility in over 95% of the simulations than placebo in the probabilistic sensitivity analysis, whereas the main analysis indicated that its utility was lower than those of amisulpride and quetiapine. A statistically significant but relatively small OR of haloperidol in the network meta-analysis may account for this result (18). This result may exemplify the limitations of statistical significance (27, 28). Sensitivity analysis using another data source showed a high utility of haloperidol in general ward settings, implying that haloperidol might also be a favorable option for delirium patients with prolonged QTc intervals. However, its utility was lower than that of placebo in PCU settings. A previous review also indicated that haloperidol could induce TdP in patients with concomitant risk factors (11). These results would require further investigation to validate the usefulness of haloperidol.

This study had several limitations. First, the data source was limited; thus, the analyses were based on only three network meta-analyses. Second, we used the data on patients with schizophrenia for the effects of antipsychotics on QTc prolongation. Third, the analyses did not include the dosage or duration of antipsychotic use. Fourth, several types of potentially effective antipsychotics, such as aripiprazole (29, 30), were not included because of the lack of data (18, 20). Fifth, the subgroup analyses could not include several drugs, such as quetiapine in PCU settings, due to the lack of data. Sixth, although we assessed the multiple patterns of utility modeling, several settings might have remained unexplored. Finally, the percentage of 36–47%, in which the superiority of quetiapine among the six treatment options and placebo was shown, might imply that quetiapine is not an absolute first-choice drug. However, given a previous study on decision analysis that concluded the superiority of a treatment strategy from the likelihood of 56–58% among seven options in the simulation (14), the likelihood of 36–47% in our study would also be unignorable.

## Conclusion

The decision analysis led us to hypothesize that quetiapine is most likely to have the highest utility for treating delirium patients with prolonged baseline QTc intervals among the six single-use antipsychotic drugs, especially in ICU settings. Haloperidol might also be useful, especially in general ward settings. However, because of the many limitations, including the lack of data concerning QTc prolongation in delirium patients, further studies are required to validate the present findings.

## Data Availability Statement

The data utilized in the present study are available in the literature (18, 20, 22). The source code used for the analysis is also available from the corresponding author upon reasonable request.

## Author Contributions

KK performed the literature search and statistical analyses. KY supervised the research project. All the authors conceived the study, participated in the interpretation of the results and writing of the report, and approved the final version.

## Conflict of Interest

The authors declare that the research was conducted in the absence of any commercial or financial relationships that could be construed as a potential conflict of interest.
