# Supplementary material for: Comparison of Antipsychotics for the Treatment of Patients With Delirium and QTc Interval Prolongation: A Clinical Decision Analysis
Source: Front Psychiatry. 2021 Jun 25;12:609678. doi: 10.3389/fpsyt.2021.609678 (PMC8267893; doi:10.3389/fpsyt.2021.609678)

Details of sensitivity analysis for the utility settings

The three parameters of the utility function (minimum utility for the status with delirium improvement, maximum utility for the status without delirium improvement, and slope) were changed as follows: (A) 30, 30, 0.05 (default); (B) 50, 30, 0.05; (C) 30, 50, 0.05; (D) 40, 40, 0.05; (E) 20, 20, 0.05; (F) 30, 30, 0.01; (G) 50, 30, 0.01; (H) 30, 50, 0.01; (I) 40, 40, 0.01; (J) 20, 20, 0.01; (K) 30, 30, 0.10; (L) 50, 30, 0.10; (M) 30, 50, 0.10; (N) 40, 40, 0.10; (O) 20, 20, 0.10.

We also prepared the following utility function: Each function had a domain of 360–600 ms (Supplementary Figure 1).

First, we used a linear function with a second derivative of 0 (Supplementary Figure 1B). The two parameters (minimum utility for the status with delirium improvement and maximum utility for the status without delirium improvement) were changed as follows: (A) 30, 30 (default); (B) 50, 30; (C) 30, 50; (D) 40, 40; (E) 20, 20.

$$\mathrm{Utility(QTc)} = \frac{(\mathrm{Min} - \mathrm{Max})}{(600 - 360)} \cdot (\mathrm{QTc} - 360) + \mathrm{Max}$$

Second, we used an exponential function with a second derivative >0 (Supplementary Figure 1C). The three parameters (minimum utility for the status with delirium improvement, maximum utility for the status without delirium improvement, and slope) were changed as follows: (A) 30, 30, 0.01 (default); (B) 50, 30, 0.01; (C) 30, 50, 0.01; (D) 40, 40, 0.01; (E) 20, 20, 0.01; (F) 30, 30, 0.005; (G) 50, 30, 0.005; (H) 30, 50, 0.005; (I) 40, 40, 0.005; (J) 20, 20, 0.005; (K) 30, 30, 0.05; (L) 50, 30, 0.05; (M) 30, 50, 0.05; (N) 40, 40, 0.05; (O) 20, 20, 0.05.

$$\mathrm{Utility(QTc)} = (\mathrm{Max} - \mathrm{Min}) \cdot \exp\left(-\mathrm{Slope} \cdot (\mathrm{QTc} - 360)\right) + \mathrm{Min}$$

Finally, we used an exponential function with a second derivative of <0 (Supplementary Figure 1D). The three parameters (minimum utility for the status with delirium improvement, maximum utility for the status without delirium improvement, and slope) were changed as follows: (A) 30, 30, 0.01 (default); (B) 50, 30, 0.01; (C) 30, 50, 0.01; (D) 40, 40, 0.01; (E) 20, 20, 0.01; (F) 30, 30, 0.005; (G) 50, 30, 0.005; (H) 30, 50, 0.005; (I) 40, 40, 0.005; (J) 20, 20, 0.005; (K) 30, 30, 0.05; (L) 50, 30, 0.05; (M) 30, 50, 0.05; (N) 40, 40, 0.05; (O) 20, 20, 0.05.

$$\mathrm{Utility(QTc)} = -(\mathrm{Max} - \mathrm{Min}) \cdot \exp\left(\mathrm{Slope} \cdot (\mathrm{QTc} - 600)\right) + \mathrm{Max}$$

Supplementary Table 1. Effect size obtained from the literature used for sensitivity analysis (20). The transition probability to the improved status was calculated using the odds ratio obtained from the literature. The transition probability of the placebo was fixed at 0.5. The plausible range corresponds to the 95% confidence interval presented in the literature.

	Transition probability to improved status (plausible range)
Intensive care unit	
Haloperidol	0.502 (0.419 – 0.590)
Quetiapine	0.889 (0.585 – 0.978)
Ziprasidone	0.552 (0.451 – 0.648)
Palliative care unit	
Haloperidol	0.412 (0.275 – 0.559)
Risperidone	0.412 (0.275 – 0.567)
General ward/medical inpatient	
Haloperidol	0.588 (0.187 – 0.900)
Olanzapine	0.435 (0.083 – 0.870)
Quetiapine	0.585 (0.213 – 0.879)
Risperidone	0.441 (0.083 – 0.873)

Supplementary Table 2. Results of probabilistic sensitivity analysis when the utility function is changed. Drugs with the largest percentage of simulations with the highest utility are shown.

Utility function	Baseline QTc intervals 420	450	480	510
Linear				
Highest utility drug	Quetiapine	Quetiapine	Quetiapine	Quetiapine
Percentage	36 %	37 %	37 %	38 %
Exponential with second derivative >0				
Highest utility drug	Quetiapine	Quetiapine	Quetiapine	Quetiapine
Percentage	39 %	37 %	35 %	34 %
Exponential with second derivative >0				
Highest utility drug	Amisulpride	Amisulpride	Quetiapine	Quetiapine
Percentage	35 %	33 %	34 %	37 %

Supplementary Table 3. Results of probabilistic sensitivity analysis when the data source is changed. The percentages of the simulations in which each drug achieved the highest utility are shown.

	Baseline QTc intervals			
Setting	420	450	480	510
Intensive care unit				
Placebo	0 %	0 %	1 %	1 %
Haloperidol	0 %	1 %	1 %	1 %
Quetiapine	98 %	98 %	98 %	98 %
Ziprasidone	2 %	1 %	0 %	0 %
Palliative care unit				
Placebo	78 %	80 %	87 %	90 %
Haloperidol	11 %	10 %	8 %	7 %
Risperidone	12 %	10 %	5 %	3 %
General ward/medical inpatient				
Placebo	4 %	4 %	6 %	7 %
Haloperidol	33 %	34 %	36 %	37 %
Olanzapine	16 %	16 %	15 %	14 %
Quetiapine	30 %	30 %	28 %	28 %
Risperidone	17 %	17 %	15 %	14 %

Supplementary Figure 1. Utility functions used in the analysis. (A) Default utility function. (B) Linear function. (C) Exponential function with a second derivative is >0. (D) Exponential function with a second derivative is <0.

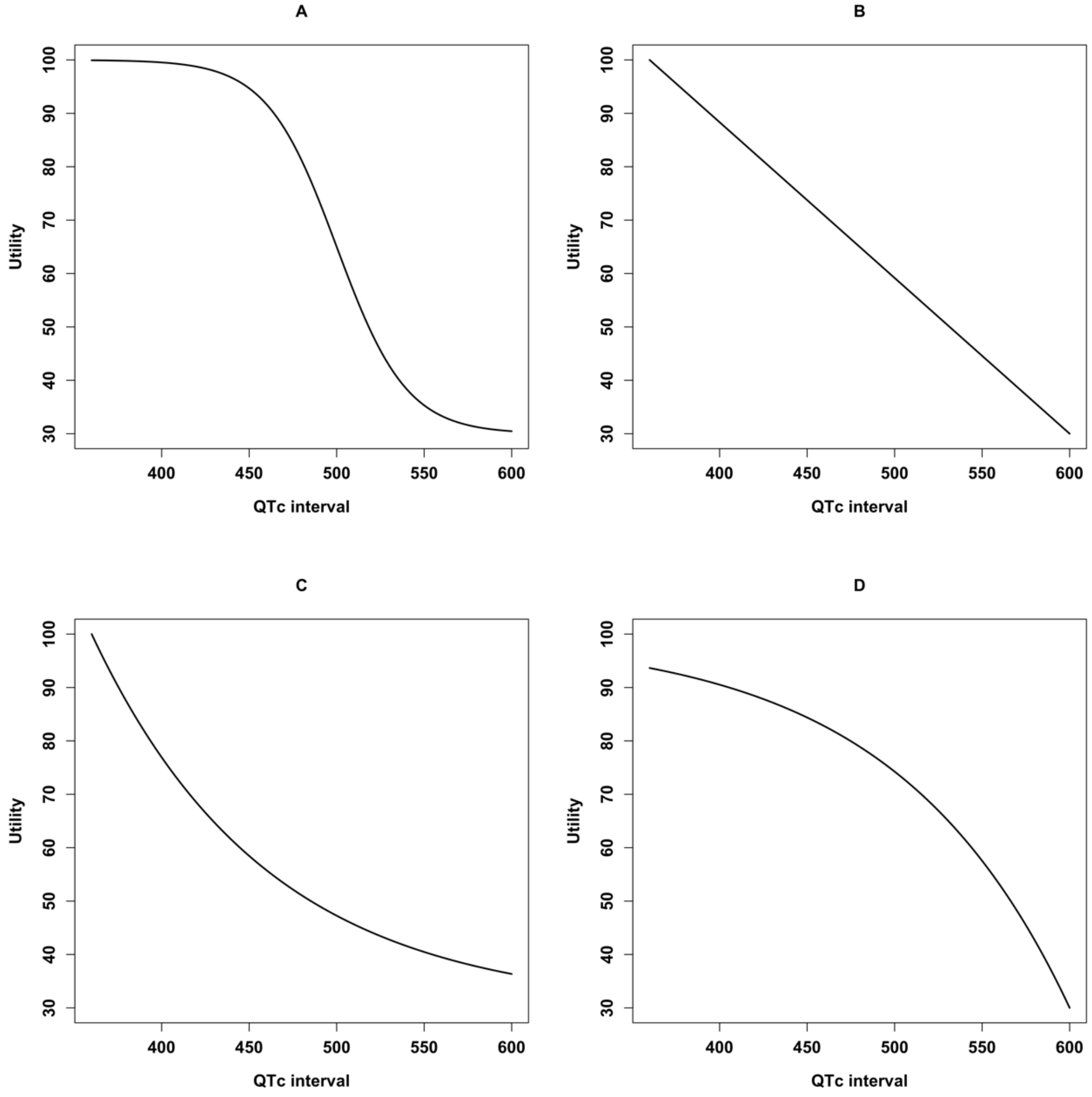

Supplementary Figure 2. Results of the sensitivity analysis. The three parameters (minimum utility for the status with delirium improvement, maximum utility for the status without delirium improvement, and slope) were as follows: (A) 30, 30, 0.05 (same as the Figure 2 in the main text); (B) 50, 30, 0.05; (C) 30, 50, 0.05; (D) 40, 40, 0.05; (E) 20, 20, 0.05; (F) 30, 30, 0.01; (G) 50, 30, 0.01; (H) 30, 50, 0.01; (I) 40, 40, 0.01; (J) 20, 20, 0.01; (K) 30, 30, 0.10; (L) 50, 30, 0.10; (M) 30, 50, 0.10; (N) 40, 40, 0.10; (O) 20, 20, 0.10. □, Placebo; ○, Amisulpride; △, Haloperidol; +, Olanzapine; ×, Quetiapine; ◇, Risperidone; ▽, Ziprasidone.

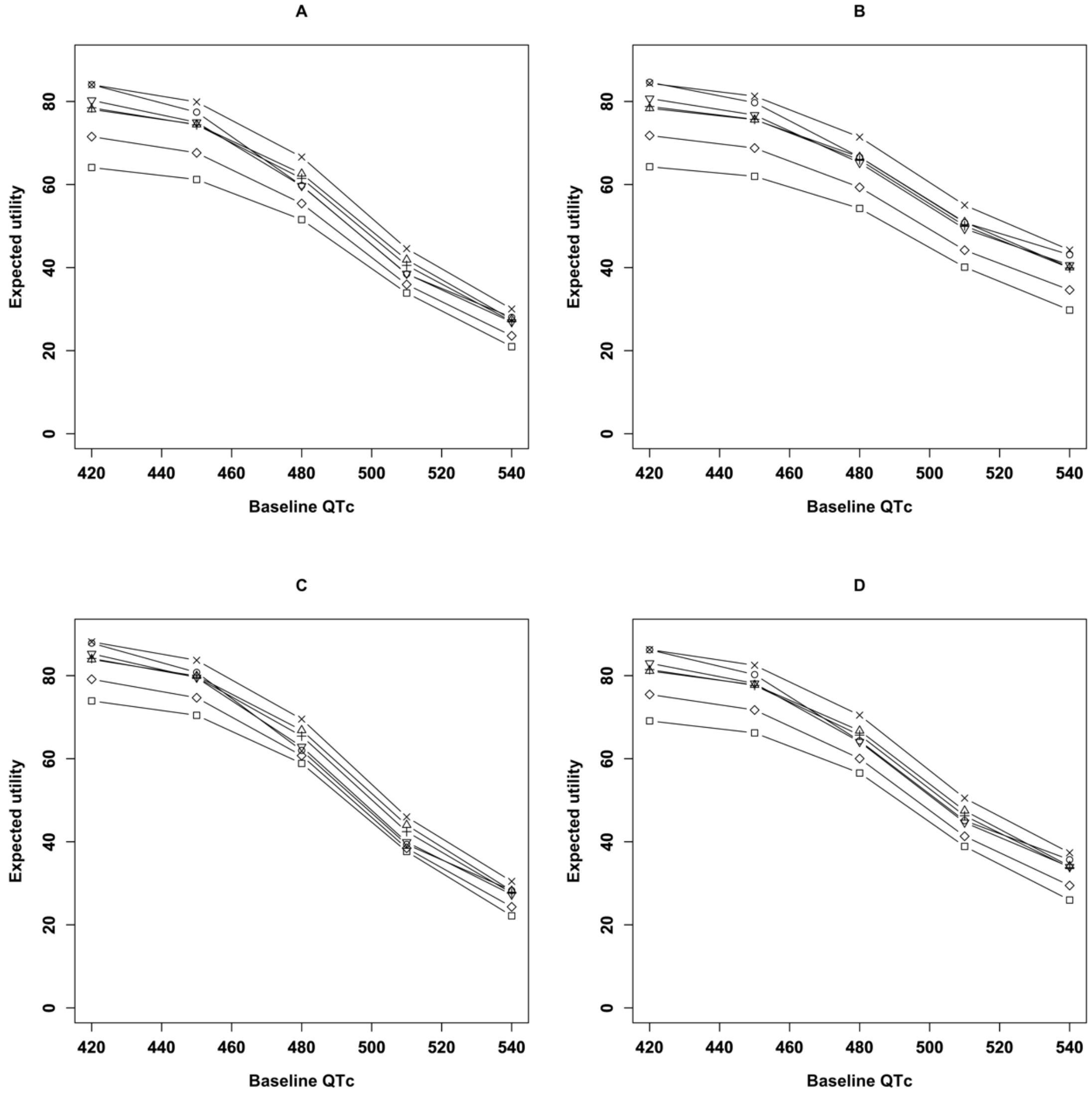

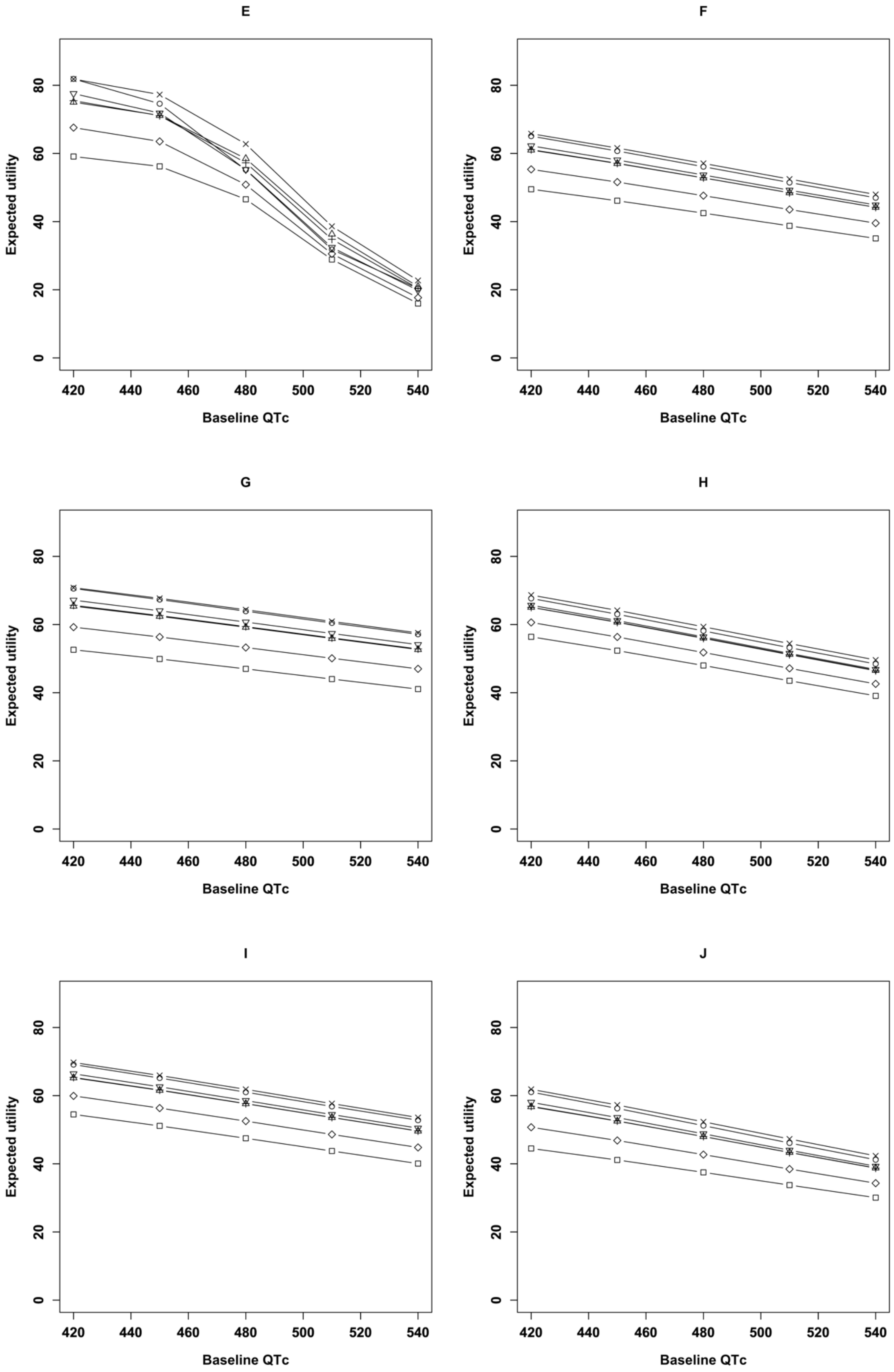
E
F
G
H
I
J
Expected utility
Baseline QTc
0
20
40
60
80
420
440
460
480
500
520
540

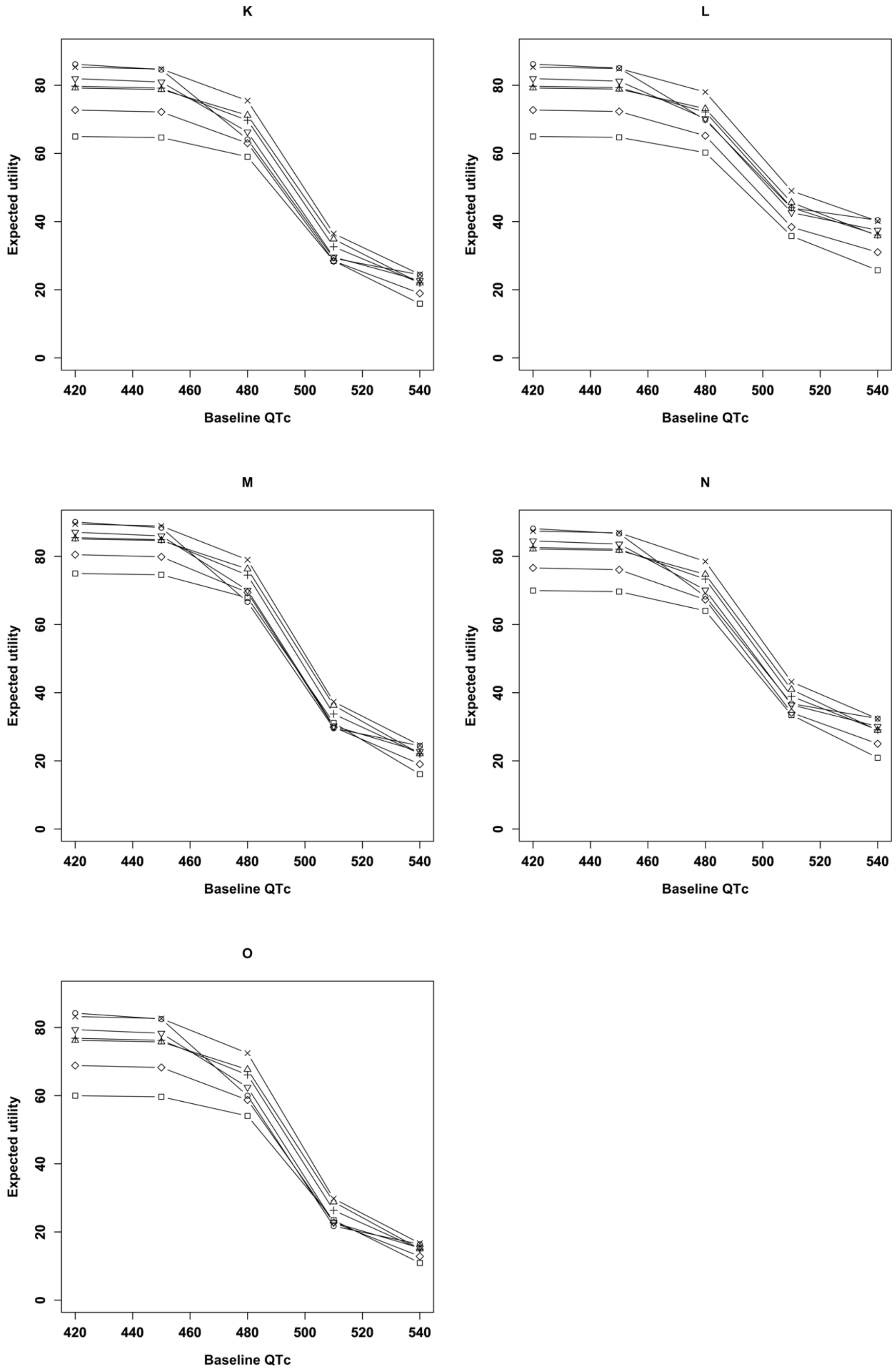
K
L
M
N
O
Expected utility
Baseline QTc
0
20
40
60
80
420
440
460
480
500
520
540

Supplementary Figure 3. Results of the sensitivity analysis using the linear function. The two parameters (minimum utility for the status with delirium improvement and maximum utility for the status without delirium improvement) were as follows: (A) 30, 30 (default); (B) 50, 30; (C) 30, 50; (D) 40, 40; (E) 20, 20. □, Placebo; ○, Amisulpride; △, Haloperidol; ＋, Olanzapine; ×, Quetiapine; ◇, Risperidone; ▽, Ziprasidone.

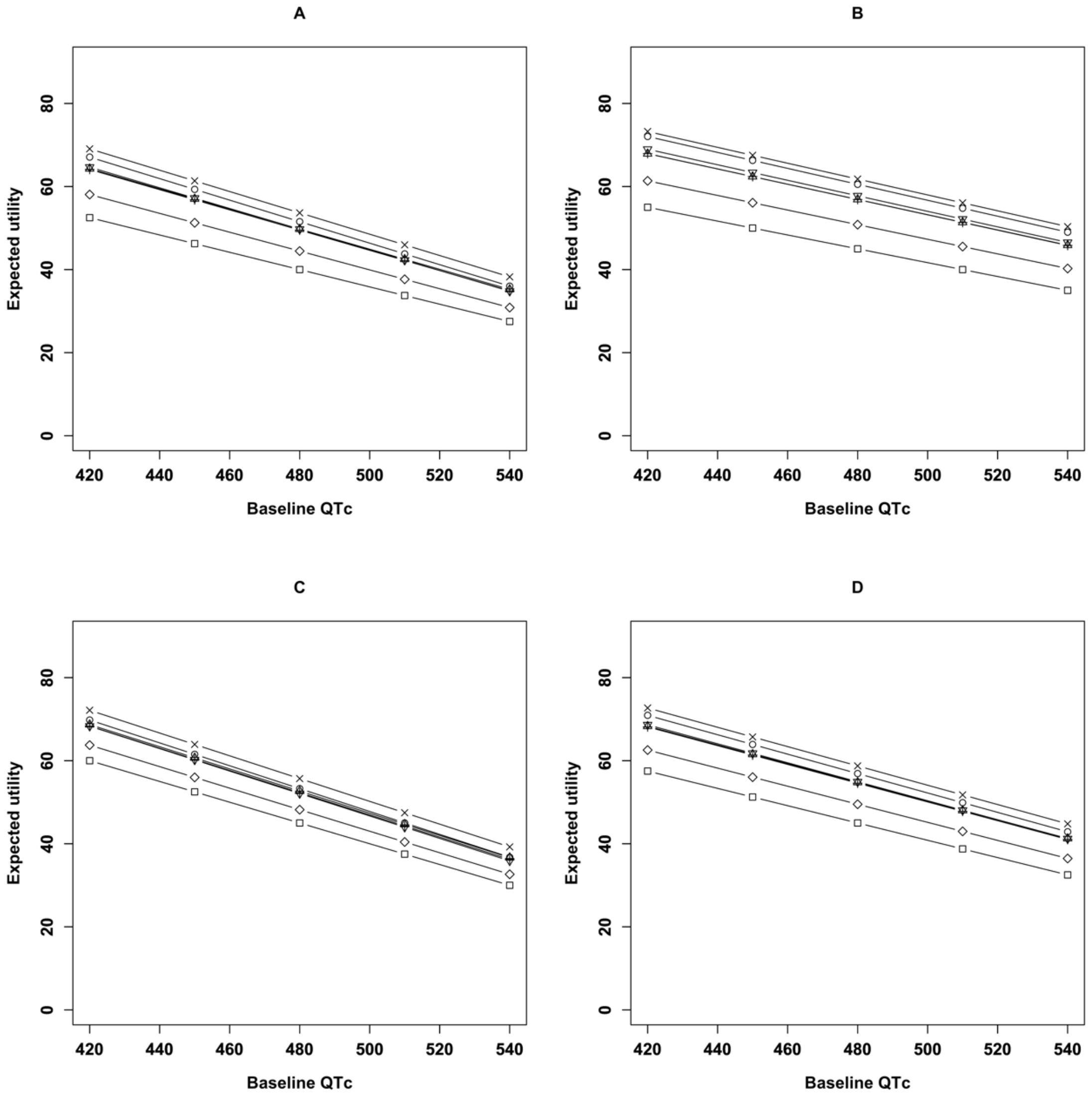

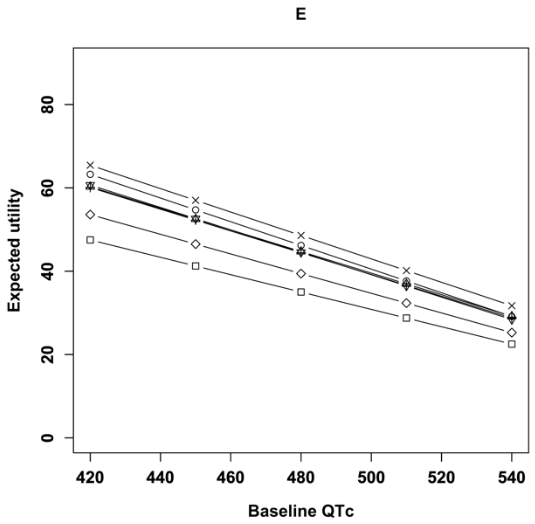
E
Expected utility
0
20
40
60
80
420
440
460
480
500
520
540
Baseline QTc

Supplementary Figure 4. Results of the sensitivity analysis using the exponential function with second derivative >0. The three parameters (minimum utility for the status with delirium improvement, maximum utility for the status without delirium improvement, and slope) were as follows: (A) 30, 30, 0.01 (default); (B) 50, 30, 0.01; (C) 30, 50, 0.01; (D) 40, 40, 0.01; (E) 20, 20, 0.01; (F) 30, 30, 0.005; (G) 50, 30, 0.005; (H) 30, 50, 0.005; (I) 40, 40, 0.005; (J) 20, 20, 0.005; (K) 30, 30, 0.05; (L) 50, 30, 0.05; (M) 30, 50, 0.05; (N) 40, 40, 0.05; (O) 20, 20, 0.05. □, Placebo; ○, Amisulpride; △, Haloperidol; +, Olanzapine; ×, Quetiapine; ◇, Risperidone; ▽, Ziprasidone.

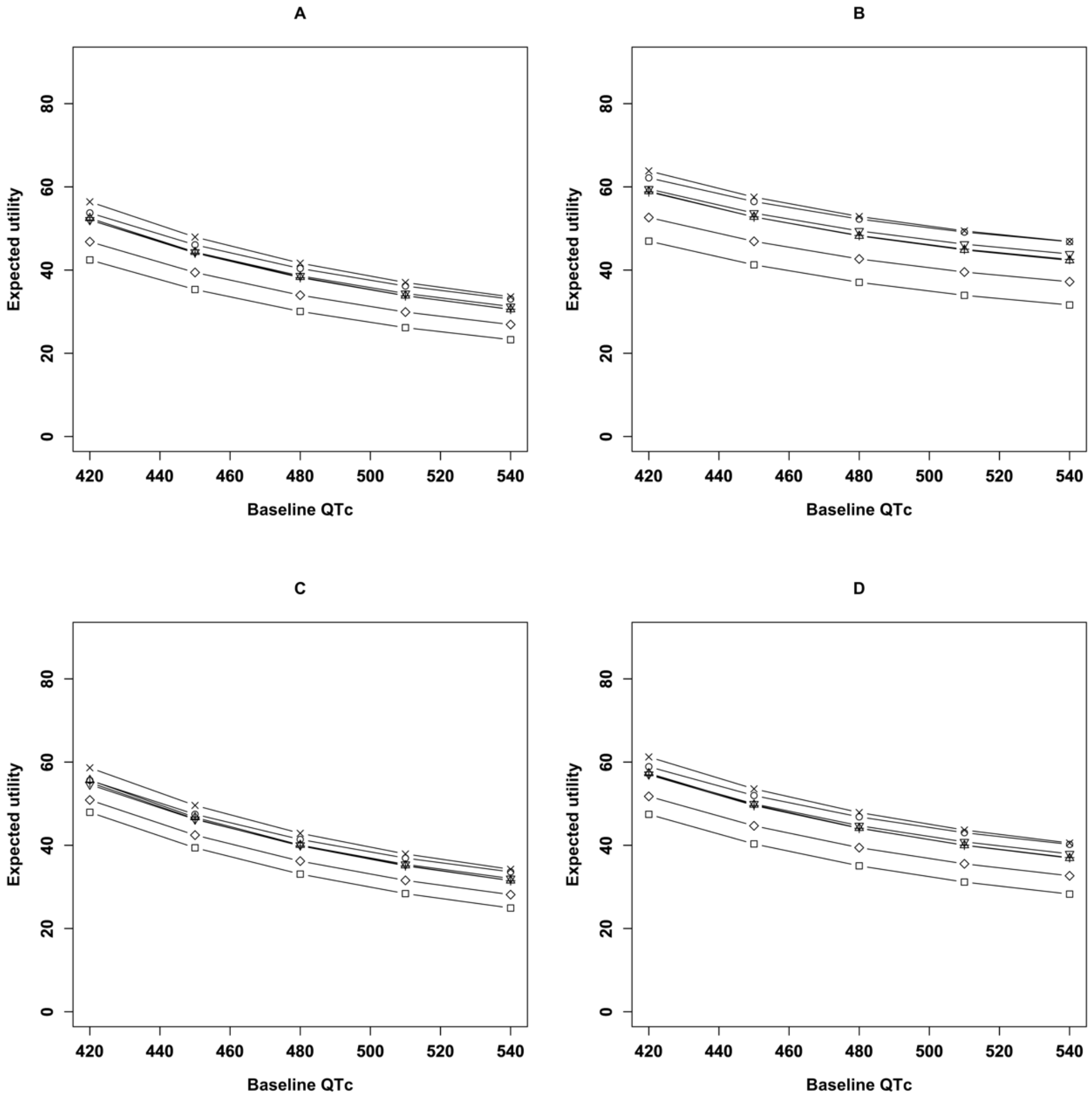

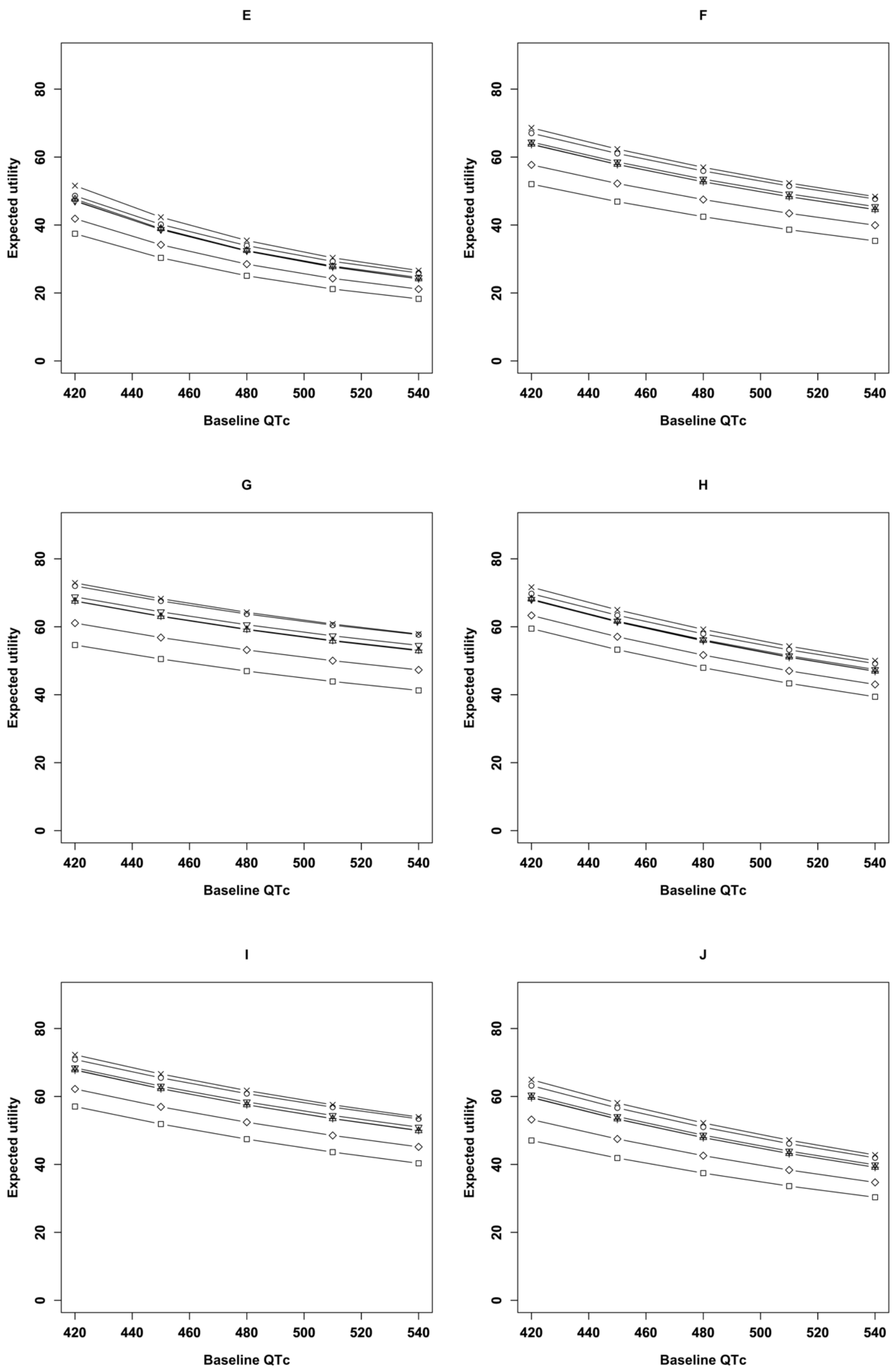
E
F
G
H
I
J
Expected utility
Baseline QTc
0
20
40
60
80
420
440
460
480
500
520
540

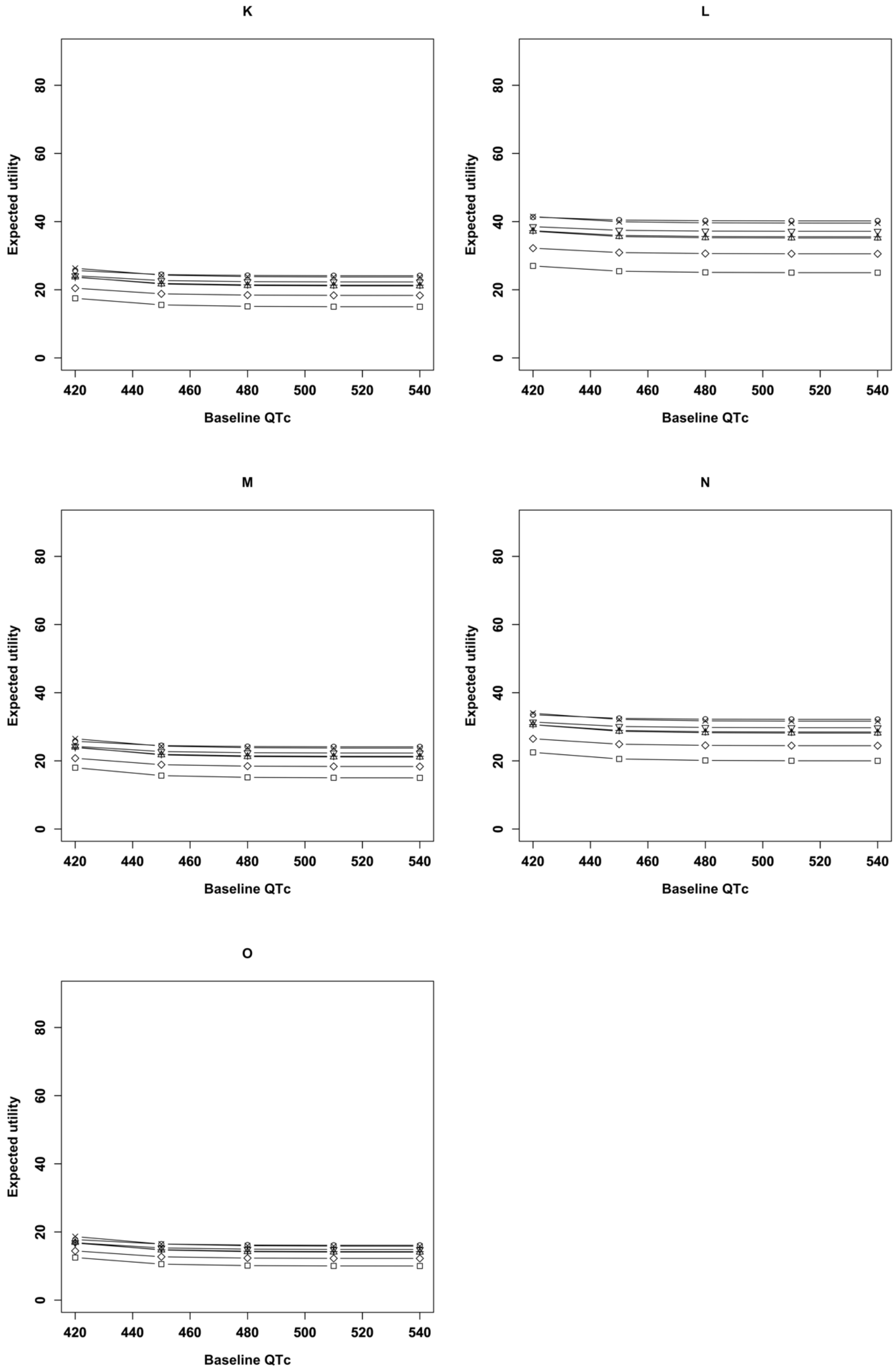
K
L
M
N
O
Expected utility
Baseline QTc
0
20
40
60
80
420
440
460
480
500
520
540

Supplementary Figure 5. Results of the sensitivity analysis using the exponential function with second derivative <0. The three parameters (minimum utility for the status with delirium improvement, maximum utility for the status without delirium improvement, and slope) were as follows: (A) 30, 30, 0.01 (default); (B) 50, 30, 0.01; (C) 30, 50, 0.01; (D) 40, 40, 0.01; (E) 20, 20, 0.01; (F) 30, 30, 0.005; (G) 50, 30, 0.005; (H) 30, 50, 0.005; (I) 40, 40, 0.005; (J) 20, 20, 0.005; (K) 30, 30, 0.05; (L) 50, 30, 0.05; (M) 30, 50, 0.05; (N) 40, 40, 0.05; (O) 20, 20, 0.05. □, Placebo; ○, Amisulpride; △, Haloperidol; ＋, Olanzapine; ×, Quetiapine; ◇, Risperidone; ▽, Ziprasidone.

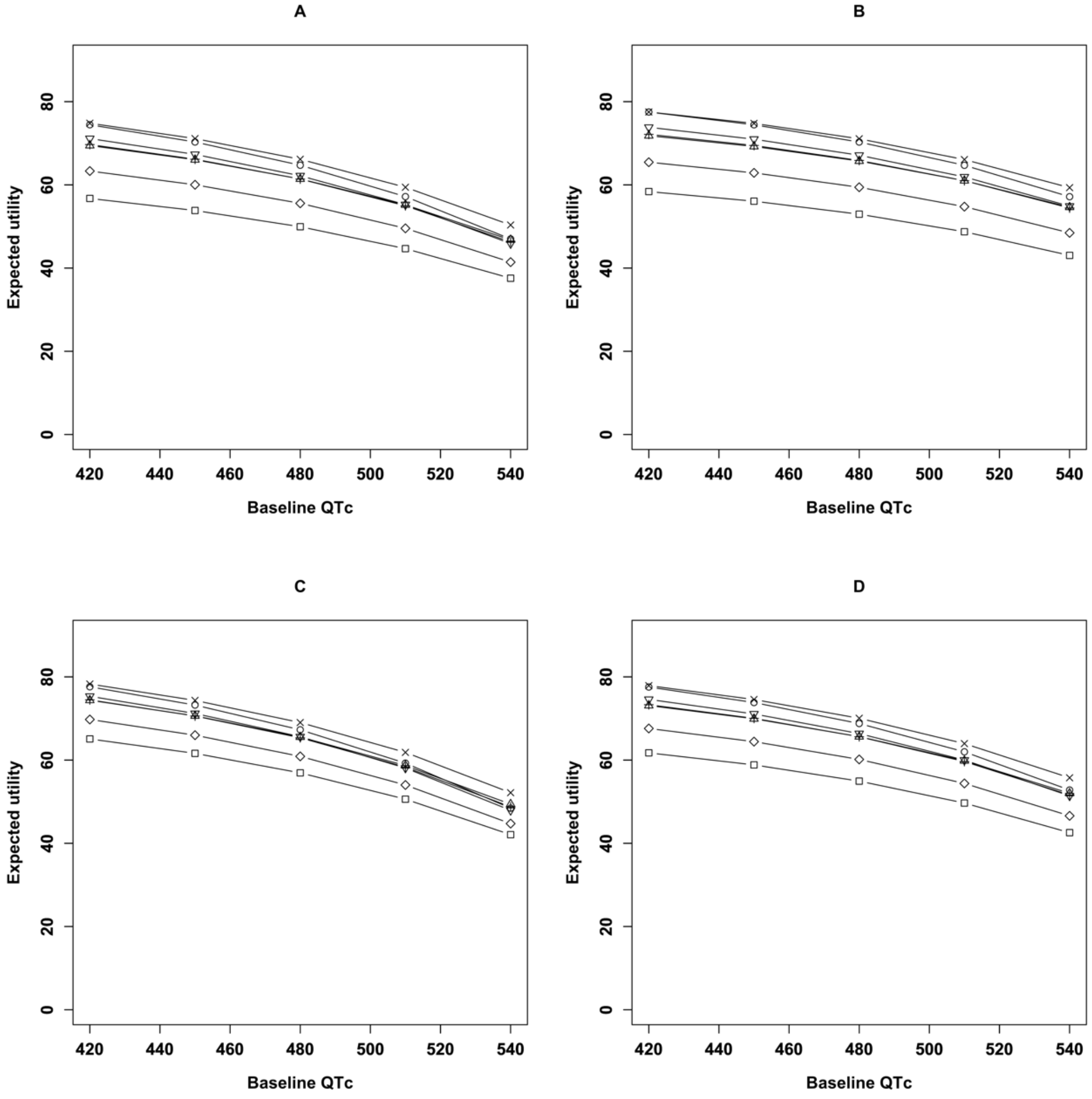

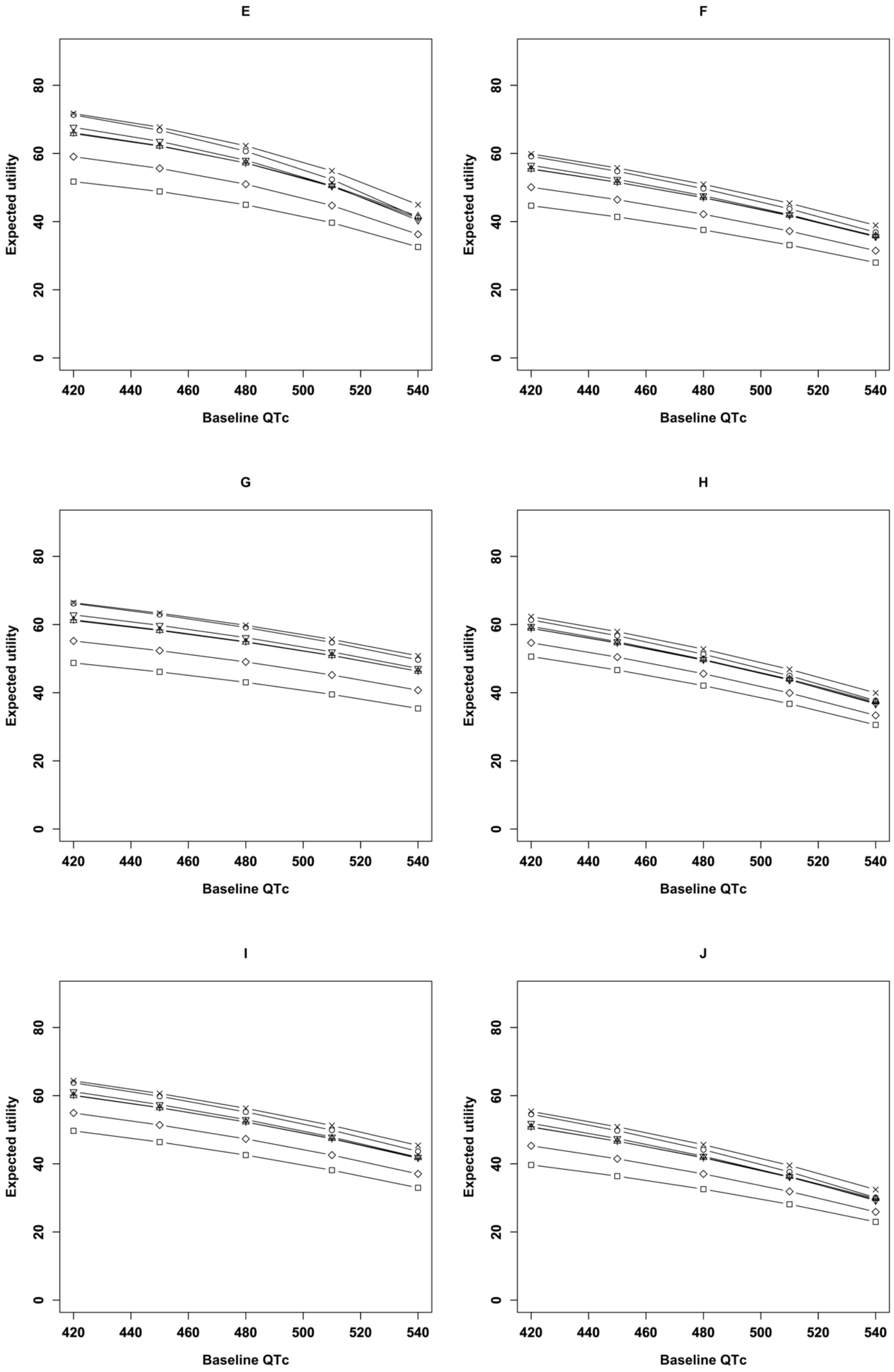
E
F
G
H
I
J
Expected utility
Baseline QTc
0
20
40
60
80
420
440
460
480
500
520
540

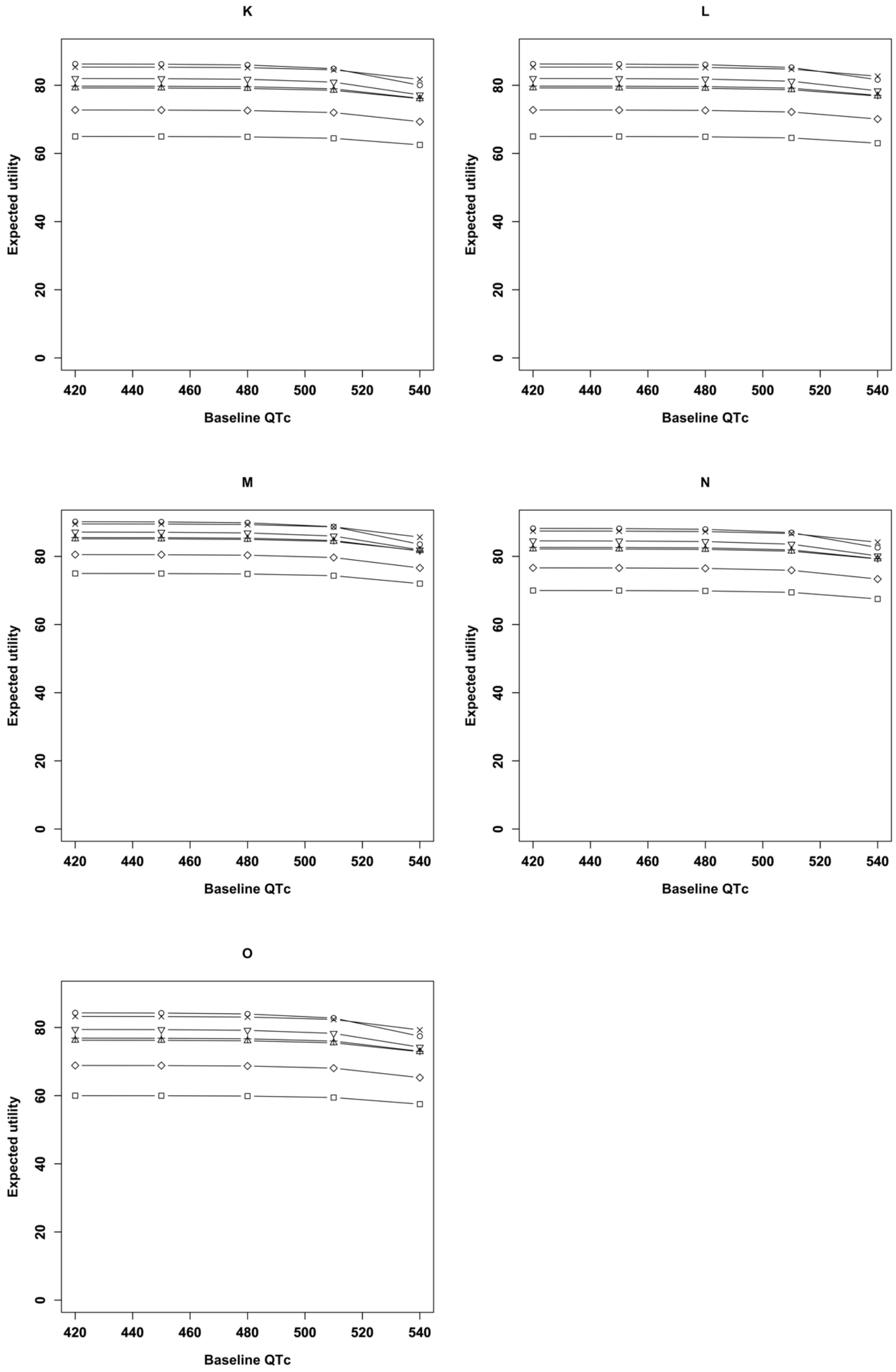
K
L
M
N
O
Expected utility
Baseline QTc
0
20
40
60
80
420
440
460
480
500
520
540

Supplementary Figure 6. Results of the sensitivity analysis using another data source. (A) Intensive care unit. (B) Palliative care unit. (C) General ward/medical inpatient. □, Placebo; △, Haloperidol; +, Olanzapine; ×, Quetiapine; ◇, Risperidone; ▽, Ziprasidone

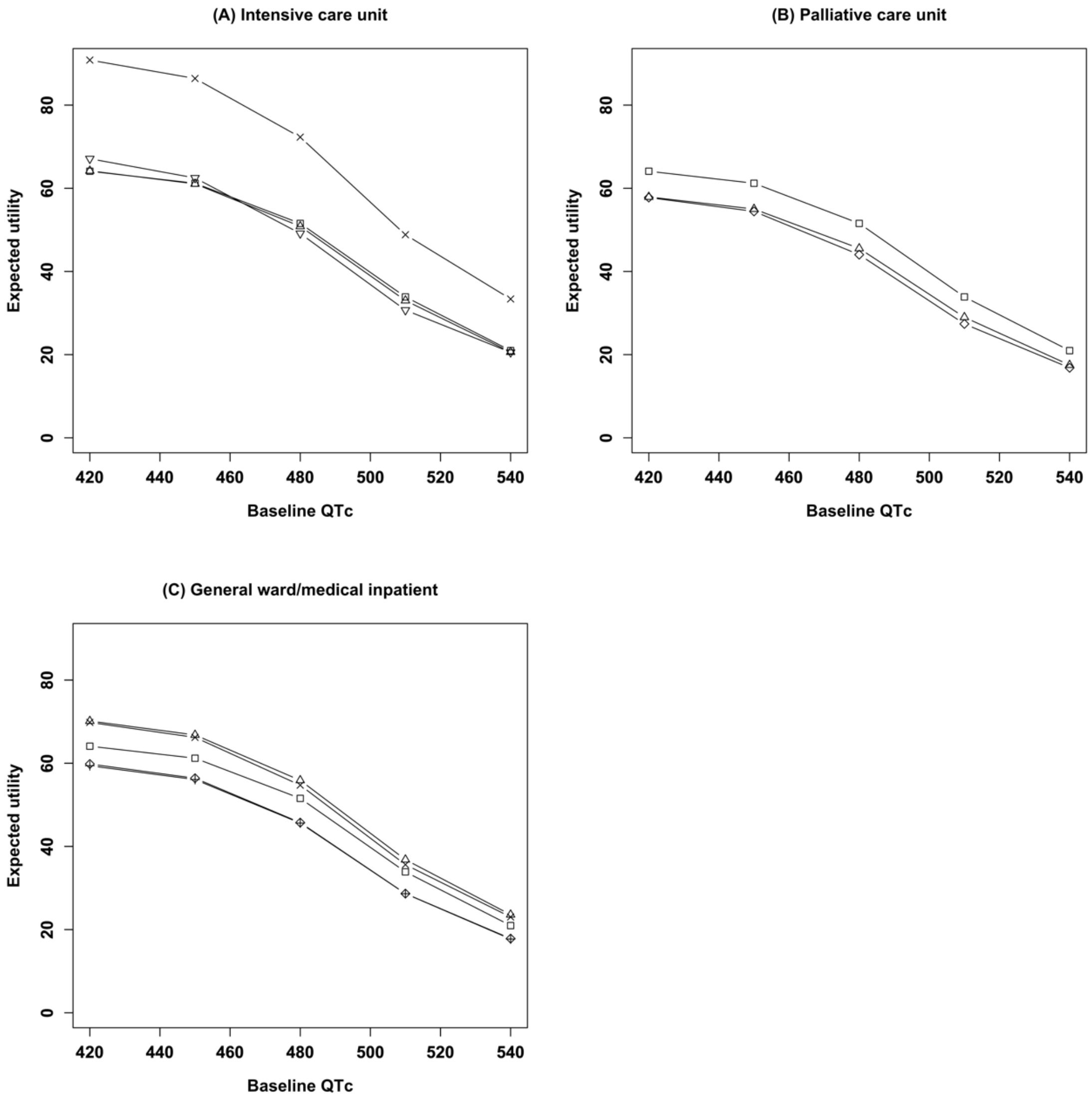

Supplement: Supplementary file 1 [file Data_Sheet_1.pdf]
